# Supplementary material for: 5-Methyl Furfural Reduces the Production of Malodors by Inhibiting Sodium l-Lactate Fermentation of Staphylococcus epidermidis: Implication for Deodorants Targeting the Fermenting Skin Microbiome
Source: Microorganisms. 2019 Aug 5;7(8):239. doi: 10.3390/microorganisms7080239 (PMC6723266; doi:10.3390/microorganisms7080239)
Supplement: Supplementary file 1 [file microorganisms-07-00239-s001.pdf]

Supplementary Materials

5-methyl Furfural Reduces the Production of Malodors by Inhibiting Sodium L-lactate Fermentation of *Staphylococcus epidermidis*: Implication for Deodorants Targeting the Fermenting Skin Microbiome

Manish Kumar [1], Binderiya Myagmardoloonjin [1], Sunita Keshari [2], Indira Putri Negari [1] and Chun-Ming Huang [1,2,3,*]

[1] Department of Biomedical Sciences and Engineering, National Central University, Taoyuan 320009, Taiwan; manishhari3@gmail.com (M.K.); binderya.smile@yahoo.com (B.M.); indiraputri24@g.ncu.edu.tw (I.P.N.)

[2] Department of Life Sciences, National Central University, Taoyuan, 320009, Taiwan; sunitakeshari827@gmail.com (S.K.)

[3] Department of Dermatology, School of Medicine, University of California, San Diego, 92093, CA, USA

* Correspondence: chunming@ncu.edu.tw

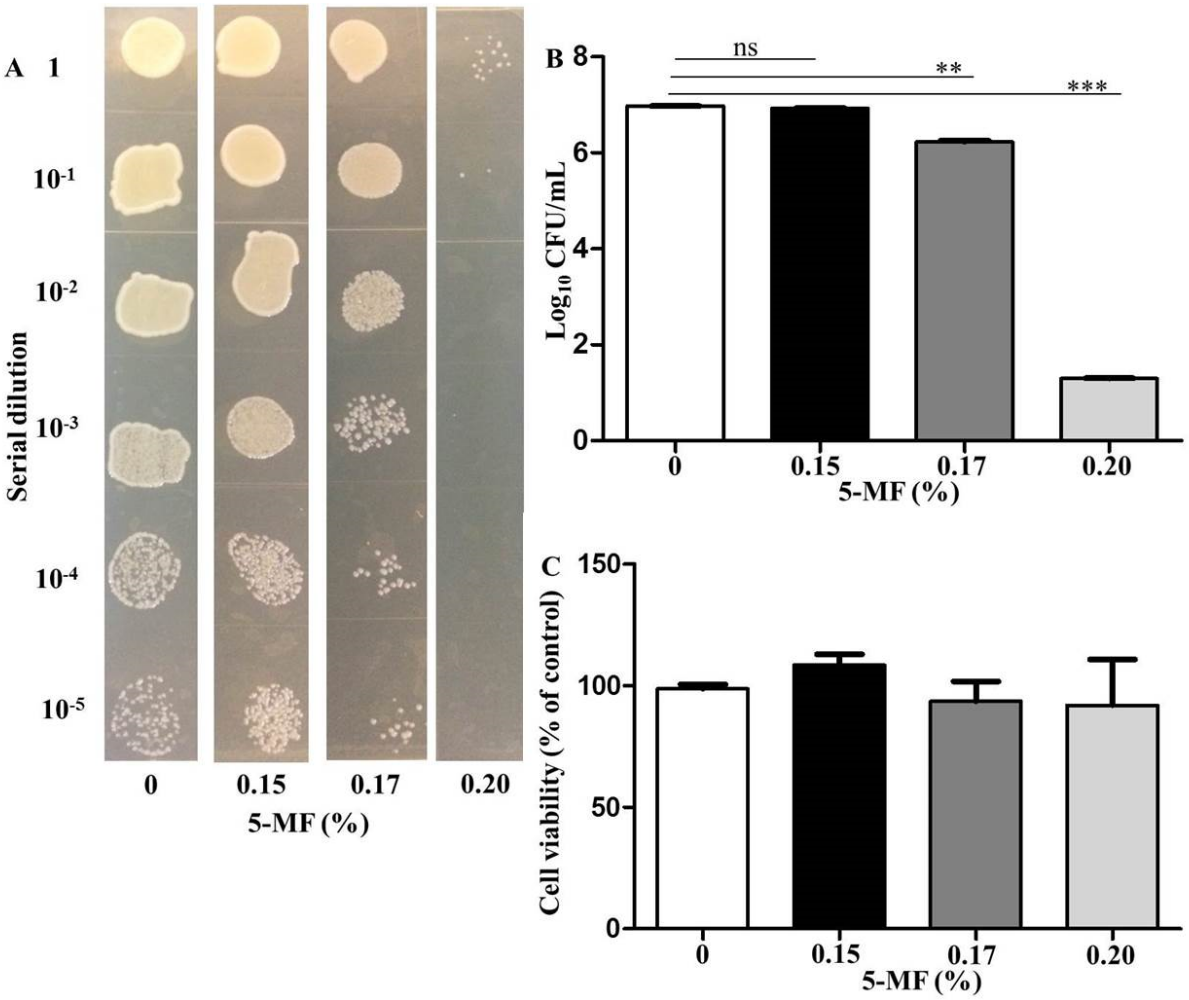

Figure S1. Effect of 5MF on the growth of *S. epidermidis* and the viability of human keratinocytes. (**A,B**) *S. epidermidis* (10^3 CFU/mL) was incubated with or without 0.15%, 0.17% or 0.20% 5MF for 24 h. CFUs (log_{10}/mL) of *S. epidermidis* were enumerated by plating serial dilutions ($1:10^1$–$1:10^5$) of culture media on a TSB plate for 24 h. (**C**) The CCD 1106 KERTr (5×10^3) keratinocyte cells, were treated with or without 0.15%, 0.17%, or 0.20% 5MF for 24 h. The viabilities of cells treated with 5MF were determined by a MTT assay and expressed as % of cells treated without 5MF (control). Data are the mean ± SD of three independent experiments. ** $p < 0.01$, *** $p < 0.001$ (two-tailed *t-tests*). ns = not significant.
